# Gut dysbiosis induces the development of asthenozoospermia through butanoate metabolism

**DOI:** 10.3389/fimmu.2026.1760881

**Published:** 2026-03-18

**Authors:** Yang Pan, Bing Li, Li Liu, Ziwei Wang, Xiaoqiang Liu

**Affiliations:** 1 Department of Gastrointestinal Surgery, The First Affiliated Hospital of Chongqing Medical University, Chongqing, China; 2 Department of Urology, Tianjin Medical University General Hospital, Tianjin, China

**Keywords:** asthenozoospermia, butanoate metabolism, fecal microbiota transplantation, gut microbiome, sperm quality

## Abstract

**Background:**

Asthenozoospermia is a leading cause of male infertility with a rising incidence. While gut dysbiosis is implicated in metabolic disease, its role in asthenozoospermia pathogenesis remains unclear.

**Materials and methods:**

We conducted a case-control study comparing the fecal microbiomes of men with isolated asthenozoospermia (n=60) and healthy controls (n=60) using shotgun metagenomic sequencing. Causality was assessed by fecal microbiota transplantation (FMT) from patients or controls into germ-free male mice. Metabolic perturbations were profiled by untargeted serum metabolomics and targeted short-chain fatty acid (SCFA) quantification in humans, alongside untargeted testicular metabolomics and serum SCFAs in recipient mice.

**Results:**

Metagenomic analysis (LEfSe) identified species-level differences, with marked depletion of butyrate-producing taxa in asthenozoospermia, most notably the prototypical butyrate producer *Faecalibacterium prausnitzii*. The relative abundance of *F. prausnitzii* was significantly positively correlated with sperm motility and progressive motility, linking gut composition to sperm quality in asthenozoospermia. Untargeted serum metabolomics identified 39 differential metabolites; KEGG enrichment prioritized butanoate metabolism. Targeted SCFA profiling confirmed significantly lower serum butyrate in asthenozoospermia versus controls. In germ-free males, FMT with patient-derived microbiota reduced sperm motility and progressive motility and induced histopathological abnormalities, including decreased interstitial Leydig cells, loss and atrophy of select intratubular cells, and an increased proportion of abnormal seminiferous tubules. Following patient FMT, recipient mice exhibited significantly reduced serum butyrate; testicular metabolomics revealed distinct profiles with 140 key differential metabolites, again implicating butanoate metabolism. Mechanistically, reduced *F. prausnitzii*-derived butyrate might impair Leydig cell steroidogenesis via disrupted PPAR signaling.

**Conclusions:**

Asthenozoospermia is associated with gut dysbiosis characterized by loss of butyrate-producing bacteria, systemic and testicular disturbances in butyrate metabolism, and microbiota-mediated transmission of impaired sperm quality. These findings implicate the gut-testis axis in asthenozoospermia pathogenesis and nominate butyrate metabolism as a potential therapeutic target.

## 1 Introduction

Male infertility affects approximately 7% of men worldwide and represents a significant public health concern with profound psychological and socioeconomic implications (1). Asthenozoospermia is characterized by reduced progressive motility of spermatozoa in semen and constitutes one of the most prevalent causes of male infertility (2). Despite substantial advances in assisted reproductive technologies, the underlying etiology of asthenozoospermia remains largely elusive, particularly in idiopathic cases that account for up to 30-40% of affected individuals (3). Established etiological factors include abnormal testosterone levels, testicular inflammation, and oxidative stress, all of which can impair spermatogenesis and sperm quality (4, 5). However, effective therapeutic interventions targeting the root causes of asthenozoospermia remain limited. Therefore, elucidating the pathogenic mechanisms underlying this condition is of paramount importance for developing safer and more efficacious treatment strategies.

Accumulating evidence indicates that sperm quality is intimately linked to systemic metabolic homeostasis (6). Specific metabolites have been shown to modulate the testicular metabolic microenvironment, influence the proliferation and differentiation of testicular cells, and thereby regulate spermatogenesis and sperm quality (7). While systemic metabolic disturbances have been implicated in impaired sperm parameters, the upstream triggers of such metabolic alterations remain poorly characterized. The gut microbiota, often referred to as the human “second genome”, participates in the development and progression of numerous diseases primarily through its metabolites (8). This vast microbial ecosystem has emerged as a critical regulator of host metabolism, immunity, and endocrine function. Marked differences in gut microbial composition have been observed between patients with primary male infertility and healthy controls, and the relative abundance of specific key taxa is significantly associated with male fertility (9). However, the role of the gut microbiota in asthenozoospermia, and whether it modulates sperm quality via its metabolites, remains unclear.

Short-chain fatty acids (SCFAs), particularly butyrate, are key metabolites produced by gut microbiota through fermentation of dietary fiber (10, 11). Biologically, butyrate differs from many other metabolites as mammalian hosts lack the enzymatic capacity for its endogenous synthesis. Consequently, the circulating butyrate pool is almost exclusively derived from the fermentation of non-digestible carbohydrates by specific anaerobic gut bacteria (12). Beyond serving as the primary energy source for colonocytes, butyrate exerts systemic effects through intestinal absorption, including modulation of immune responses, maintenance of epithelial barrier integrity, and regulation of metabolic processes (12, 13). However, whether gut-derived butyrate can cross the blood-testis barrier to influence testicular steroidogenesis remains to be elucidated. Delineating the potential roles of the gut microbiota and its metabolites in the pathogenesis and treatment of asthenozoospermia is of substantial clinical significance and may provide new therapeutic avenues for male infertility.

This study aimed to elucidate whether gut microbial dysbiosis contributes to the development of asthenozoospermia through alterations in SCFA metabolism. We hypothesized that gut dysbiosis reduces butyrate-producing bacteria, leading to decreased circulating butyrate levels, impaired Leydig cell steroidogenesis, reduced testosterone production, and ultimately diminished sperm motility. To test this hypothesis, we conducted a comprehensive investigation integrating human cohort analyses, fecal microbiota transplantation (FMT) experiments in germ-free mice, multi-omics profiling, and metabolite rescue studies in model mice. Our findings establish a causal link between gut dysbiosis and asthenozoospermia and identify the gut-butyrate-testis axis as a novel therapeutic target for male infertility.

## 2 Materials and methods

### 2.1 Study subjects

#### 2.1.1 Inclusion and exclusion criteria

We recruited patients with asthenozoospermia and healthy controls through the health examination center and fertility counseling platforms. A standardized questionnaire was developed to capture basic information, dietary habits, lifestyle factors, family history, and occupation, with measures to ensure truthful completion and enhance data accuracy. Inclusion criteria were as follows: 1) Men aged 20-40 years with a BMI of 18-24 kg/m²; 2) Similar dietary habits, avoiding extreme diets; 3) Patients with isolated asthenozoospermia, without other reproductive abnormalities. Exclusion criteria were as follows: 1) Known infections, endocrine disorders, or other diseases or surgical histories that affect reproduction; 2) Other clinical symptoms or conditions that may influence gut microbiota assessments, such as depression or inflammatory bowel disease; 3) Use of hormones, antibiotics, probiotics, or prebiotics within the past month. Detailed exclusion criteria also included history of heavy smoking (>10 cigarettes/day) or alcohol consumption (>2 drinks/week), and recent exposure to environmental toxins. The control group consisted of men with normozoospermia and proven fertility (conception within the last 2 years).

#### 2.1.2 Ethics statement

The study protocol for human cohorts was approved by the institutional review board of Tianjin Medical University General Hospital. Informed consent was confirmed by the Institutional Review Board (IRB). The animal studies were performed after receiving approval of the Institutional Animal Care and Use Committee (IACUC) in Tianjin Medical University. The work has been reported in accordance with the ARRIVE (Animals in Research: Reporting In Vivo Experiments) guidelines (14).

#### 2.1.3 Human semen analysis

Semen collection and analysis were conducted in accordance with the standardized procedures outlined in the World Health Organization (WHO) Laboratory Manual for the Examination and Processing of Human Semen, sixth edition (15). All participants observed 3-5 days of sexual abstinence and provided semen samples by masturbation. Semen parameters were measured and analyzed using a computer-assisted sperm analysis (CASA) system.

### 2.2 Shotgun metagenomic sequencing

Each participant provided a single fresh stool sample for shotgun metagenomic sequencing. Total microbial DNA was extracted using the Cetyltrimethylammonium bromide (CTAB) method. DNA extraction quality was assessed by agarose gel electrophoresis. DNA concentration was quantified with an ultraviolet (UV) spectrophotometer. Shotgun metagenomic sequencing was performed on the Illumina NovaSeq 6000 platform as 2 x 150 bp paired-end reads. The detailed materials and analysis methods were described in the **Supplementary Material** (**Supplementary Materials and Methods 1**).

### 2.3 Untargeted metabolomics analysis

Analyses were performed using an UHPLC (Vanquish, Thermo Fisher Scientific) coupled to Q Exactive HFX mass spectrometer (Orbitrap MS, Thermo). The detailed materials and analysis methods were described in the **Supplementary Material** (**Supplementary Materials and Methods 2**).

### 2.4 Targeted SCFAs metabolomics analysis

Serum was collected using sterile single-use venipuncture needles, separated by centrifugation, and analyzed for SCFAs by targeted liquid chromatography-mass spectrometry (LC-MS/MS). The detailed materials and analysis methods were described in the **Supplementary Material** (**Supplementary Materials and Methods 3**).

### 2.5 Fecal microbiota transplantation

Fecal samples were collected from patients with asthenozoospermia and healthy controls. Fecal samples from donors were pooled to create a standardized inoculum to minimize individual variation. A defined amount was weighed into sterile grinding tubes. For each gram of feces, 4 mL of sterile phosphate-buffered saline (PBS) was added, and the mixture was thoroughly homogenized to obtain a uniform suspension. The homogenate was passed through a 200−mesh sterile filter to remove large particulates and debris. Sterile glycerol was added to the filtrate to a final concentration of 20% (v/v) and mixed thoroughly to ensure uniformity. The prepared suspension was aliquoted into sterile cryovials at 2 mL per tube, labeled, and stored at -80 °C for subsequent use in FMT experiments.

Germ-free (GF) male C57BL/6J mice (8 weeks old) were purchased from GemPharmatech Co., Ltd. (Nanjing, China). The mice were maintained in sterile flexible-film plastic isolators at the Animal Center. They were kept under a 12-hour light/dark cycle at 23 ± 2°C and provided with autoclaved water and sterile vacuum-packed chow ad libitum. Male germ-free mice were randomly assigned to two groups: the asthenozoospermia FMT group (AS-FMT), which received bacterial suspensions derived from patients with asthenozoospermia, and the normal control FMT group (NC-FMT), which received suspensions derived from healthy controls. Using aseptic technique, 250 µL of the prepared bacterial suspension was withdrawn for each administration and delivered to recipient mice by oral gavage. FMT was performed every other day for 8 weeks to ensure stable microbiota engraftment.

Gut microbiota profiling in recipient mice was analyzed by metagenomic sequencing to demonstrate successful engraftment and replication of donor microbial profiles. To validate the relative abundance changes observed in the sequencing data, the abundance of F. prausnitzii was quantified using quantitative real-time PCR (qPCR). The same fecal DNA samples used for sequencing served as templates. qPCR was performed on a CFX96 Real-Time PCR Detection System (Bio-Rad, Hercules, CA, USA) using SYBR Green qPCR Master Mix (Takara, Tokyo, Japan). Specific primers for F. prausnitzii were used, and the total bacterial load (Universal 16S rRNA) served as the internal control for normalization. The relative abundance was calculated using the 2−ΔΔCt method. The primer sequences used in this study are listed below: F. prausnitzii (Forward: 5’-CCATGAATTGCCTTCAAAACTGTT-3’; Reverse: 5’-GAGCCTCAGCGTCAGTTGGT-3’) Universal 16S rRNA (Total Bacteria) (Forward: 5’-ACTCCTACGGGAGGCAGCAG-3’; Reverse: 5’-ATTACCGCGGCTGCTGG-3’). The relative abundance of F. prausnitzii was calculated using the 2-ΔΔCt method.

Sodium butyrate (Sigma-Aldrich) was dissolved in sterile water. Mice in the specific groups received sodium butyrate via oral gavage at a dose of 500 mg/kg body weight daily for 8 weeks.

### 2.6 Single-cell RNA sequencing of testicular tissue

Single-cell RNA sequencing (scRNA-seq) of testicular tissue involves the isolation of individual testicular cells, followed by high-throughput sequencing to analyze their transcriptomic profiles. Briefly, fresh testicular tissue is enzymatically dissociated into a single-cell suspension, filtered, and viability-checked before library preparation using a microfluidic platform. After cDNA synthesis and amplification, libraries are sequenced to obtain transcriptomic data for each cell. Downstream bioinformatic analyses include quality control, normalization, dimensionality reduction, clustering, and identification of cell types based on marker gene expression. Further analyses such as differential expression and cell trajectory inference are performed to investigate spermatogenic lineage differentiation and testicular microenvironmental regulation. The detailed materials and analysis methods were described in the **Supplementary Material** (**Supplementary Materials and Methods 4**).

### 2.7 RNA isolation and qPCR

Total RNA was extracted using the Trizol reagent according to the manufacturer’s instructions. Extracted RNA was dissolved in 10 μL RNase-free water, then measured at 260/280 nm and stored at -80°C prior to use. cDNA was prepared from 1 μg of total RNA in a 10 μL reaction mixture containing random primers following a standard protocol. Real-time PCR was performed using the validated SYBR Green gene expression assay in combination with the SYBR Premix Ex Taq II for measuring Hsd3b1, Cyp17a1, Star, Cyp19a1, Acbd3, Hsd17b3, and Gapdh. All the primers used in this research are listed in **Supplementary Material** (**Supplementary Table 1**). Quantitative real-time PCR was performed in Stepone Plus (Thermo Fisher Scientific). The comparative Ct method standardized to GAPDH was used to quantify gene expression levels.

### 2.8 Mouse sperm quality and hormone assessment

Following completion of microbiota transplantation, mice were anesthetized. The epididymides were excised and rinsed with sterile PBS. The cauda epididymis was isolated and placed into a microcentrifuge (EP) tube containing 1 mL of prewarmed 37 °C PBS. The cauda was finely minced in the saline, and the tube was incubated in a 37 °C water bath for 10 minutes to allow sperm to be released into the medium. After incubation, a 10 μL aliquot of the sperm suspension was loaded onto a pre-warmed (37°C) CASA counting chamber (depth 20 μm, Leja), and sperm concentration and motility parameters were analyzed using CASA system. To minimize algorithmic bias, CASA results were validated by manual counting performed by an experienced technician blinded to the group allocation. Serum testosterone levels were quantified using a commercial ELISA kit (Mlbio Biotechnology, Shanghai, China) following the manufacturer’s instructions.

### 2.9 Functional analysis of mouse testis

Thin sections of mouse testis were prepared and stained with H&E to observe structural alterations and to evaluate changes in spermatogenesis and sperm maturation. To evaluate spermatogenesis, the seminiferous tubules were assessed using Johnsen’s mean testicular biopsy score (MTBS), ranging from 1 (no cells in tubular section) to 10 (complete spermatogenesis with many spermatozoa). Fifty seminiferous tubules were randomly analyzed per biological replicate in a blinded manner. For Leydig cell quantification, five random high-power fields (400×) were selected for each mouse. The number of cells with typical Leydig morphology (round nuclei and eosinophilic cytoplasm) in the interstitial space was counted.

Immunohistochemical staining for proliferating cell nuclear antigen (PCNA) in testicular tissue is performed to evaluate cell proliferation activity. Paraffin-embedded testicular sections are deparaffinized, rehydrated, and subjected to antigen retrieval using citrate buffer (pH 6.0) under controlled heating conditions. After blocking endogenous peroxidase activity and nonspecific binding, the slides are incubated with an anti-PCNA primary antibody at an optimized dilution, followed by a species-specific secondary antibody conjugated to horseradish peroxidase (HRP). Visualization is achieved with a DAB chromogen, and sections are counterstained with hematoxylin, dehydrated, and mounted. Positive PCNA immunoreactivity appears as brown nuclear staining in proliferating germ and somatic cells. For quantitative analysis, the PCNA labeling index is determined by calculating the percentage of positively stained nuclei among total counted cells in randomly selected high-power microscopic fields.

Immunofluorescence staining was applied for Leydig Cell quantification. Testicular tissues were collected and fixed in 4% paraformaldehyde (PFA) for 24 hours, dehydrated through a graded ethanol series, and embedded in paraffin. Tissue sections (5 μm thick) were deparaffinized in xylene and rehydrated. Antigen retrieval was performed by heating the sections in citrate buffer (pH 6.0) in a microwave for 15 min. After cooling to room temperature, the sections were washed with PBS and blocked with 5% bovine serum albumin (BSA) containing 0.3% Triton X-100 for 1 hour at room temperature to prevent non-specific binding. The sections were then incubated overnight at 4 °C with a primary antibody against HSD3B1 (1:200; BOSTER, China). Following three washes with PBS, the sections were incubated with an Alexa Fluor 488-conjugated secondary antibody (1:500) for 1 hour at room temperature in the dark. Nuclei were counterstained with DAPI (4’,6-diamidino-2-phenylindole). Images were captured using a Zeiss LSM 880 fluorescence microscope. For the quantification of Leydig cells, at least five non-overlapping fields were randomly selected from each testicular section. The number of HSD3B1-positive cells (green) in the interstitial space was manually counted using ImageJ software (National Institutes of Health, USA). The data were expressed as the average number of Leydig cells per field of view.

### 2.10 Data and statistical analysis

According to the different data, statistical analysis between groups were analyzed using the Wilcoxon’s rank-sum test, Student’s t-test. Correlation analyses were performed based on the Spearman’s rho statistic. Rate comparisons were performed with Pearson’s χ2 test or Fisher’s exact test. Multiple hypotheses were adjusted using the Benjamini and Hochberg method. Statistical analysis was performed using GraphPad Prism (v9.0) or R (v4.1.0). The normality of data was assessed using the Shapiro-Wilk test. For comparison between two groups, an unpaired two-tailed Student’s t-test was used. For multiple group comparisons, one-way ANOVA followed by Tukey’s post-hoc test was applied. A two tailed P-value <0.05 was considered statistically significant.

## 3 Results

### 3.1 Differences in the gut microbiota between patients with asthenozoospermia and healthy controls

**Figure 1:**
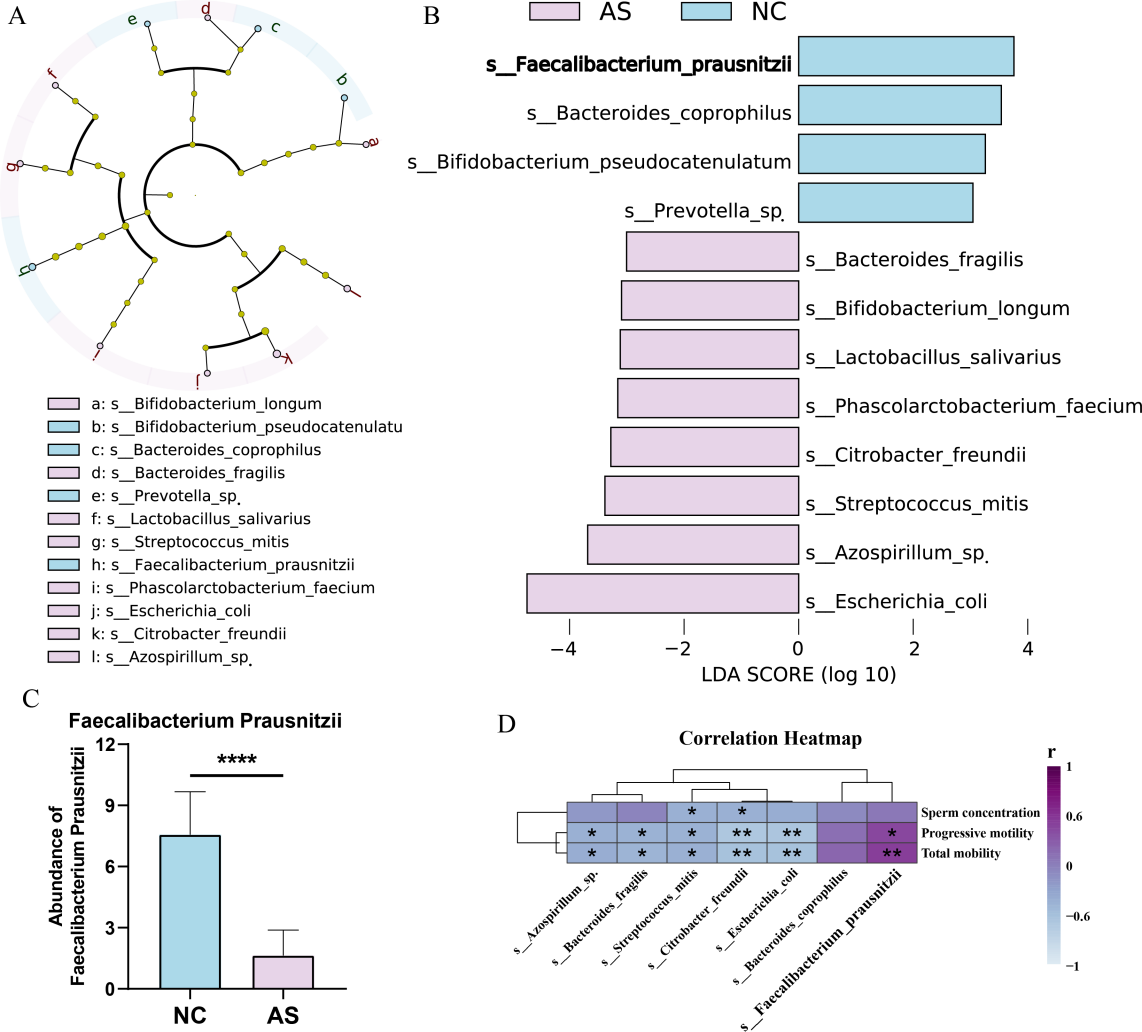
Gut microbiota profiling in patients with asthenozoospermia and healthy controls. **(A)** Phylogenetic cladogram generated by LEfSe (Linear discriminant analysis Effect Size); **(B)** Bar plot of LDA scores showing taxa with LDA > 3 and P < 0.05; **(C)** Comparison of the relative abundance of *Faecalibacterium prausnitzii* between groups; **(D)** Clustered heatmap of correlations between key differential taxa and sperm quality parameters. NC, healthy control group; AS, asthenozoospermia group. *, P<0.05; **, P<0.01; ****, P<0.0001.

A total of 60 patients with isolated asthenozoospermia and 60 healthy control men were included in the present study. The gut microbiota analysis was performed to characterize species-level features, and LEfSe (linear discriminant analysis effect size) identified species-level differentially abundant taxa between groups (**Figures 1A, B**). Compared with healthy controls, butyrate-producing taxa were significantly depleted in patients with asthenozoospermia, most notably a marked reduction in the abundance of the prototypical butyrate producer F. prausnitzii (**Figure 1C**). The correlation analysis also showed that the relative abundance of F. prausnitzii was significantly positively correlated with sperm motility and progressive motility (**Figure 1D**), revealing a relationship between the gut microbiota and sperm quality in asthenozoospermia.

### 3.2 Serum butyrate levels are significantly lower in patients with asthenozoospermia than in healthy controls

**Figure 2:**
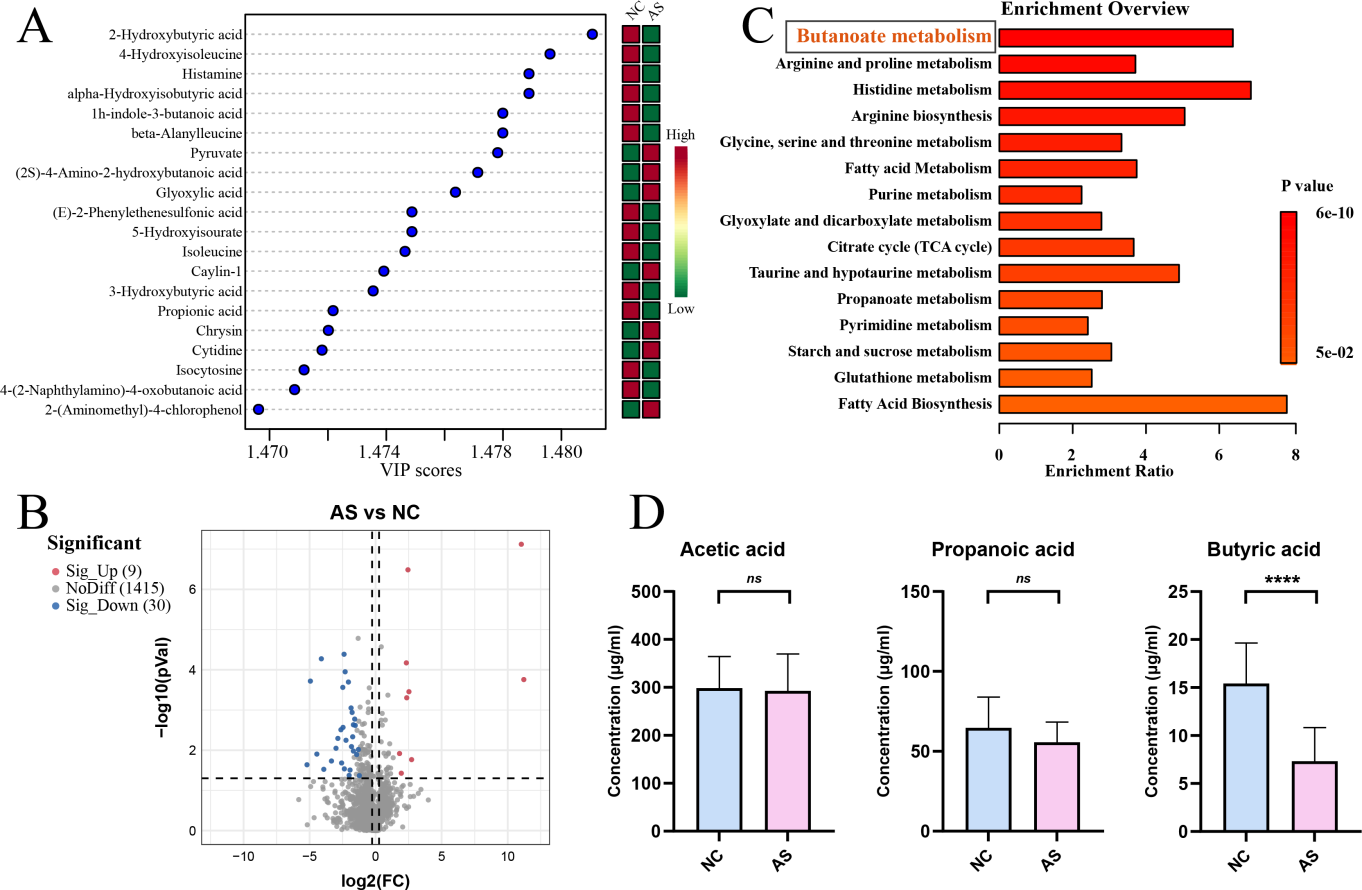
Serum metabolomic profiling in patients with asthenozoospermia and healthy controls. **(A)** Top 20 variable importance in projection **(VIP)** scores for differential metabolites from untargeted serum metabolomics; **(B)** Volcano plot of differential metabolites; **(C)** KEGG pathway enrichment analysis of differential metabolites, showing significant enrichment of the butyrate metabolism pathway; **(D)** Targeted quantification of serum short-chain fatty acids (SCFAs), demonstrating a significant reduction in butyrate levels in the asthenozoospermia group. NC, healthy control group; AS, asthenozoospermia group. ****, P<0.0001.

We conducted untargeted metabolomic profiling of serum from patients with asthenozoospermia and healthy controls, where orthogonal partial least squares–discriminant analysis (OPLS−DA) combined with differential expression analysis identified 39 differential metabolites (**Figures 2A, B**). KEGG functional enrichment using MetaboAnalyst 6.0 revealed that the butanoate metabolism pathway was significantly enriched and ranked highest (**Figure 2C**). Subsequently, targeted metabolomics of serum short-chain fatty acids showed that serum butyrate levels were significantly reduced in patients with asthenozoospermia compared with healthy controls (**Figure 2D**).

### 3.3 Fecal microbiota transplantation confirms that microbiota from patients with asthenozoospermia lowers sperm quality in male mice

**Figure 3:**
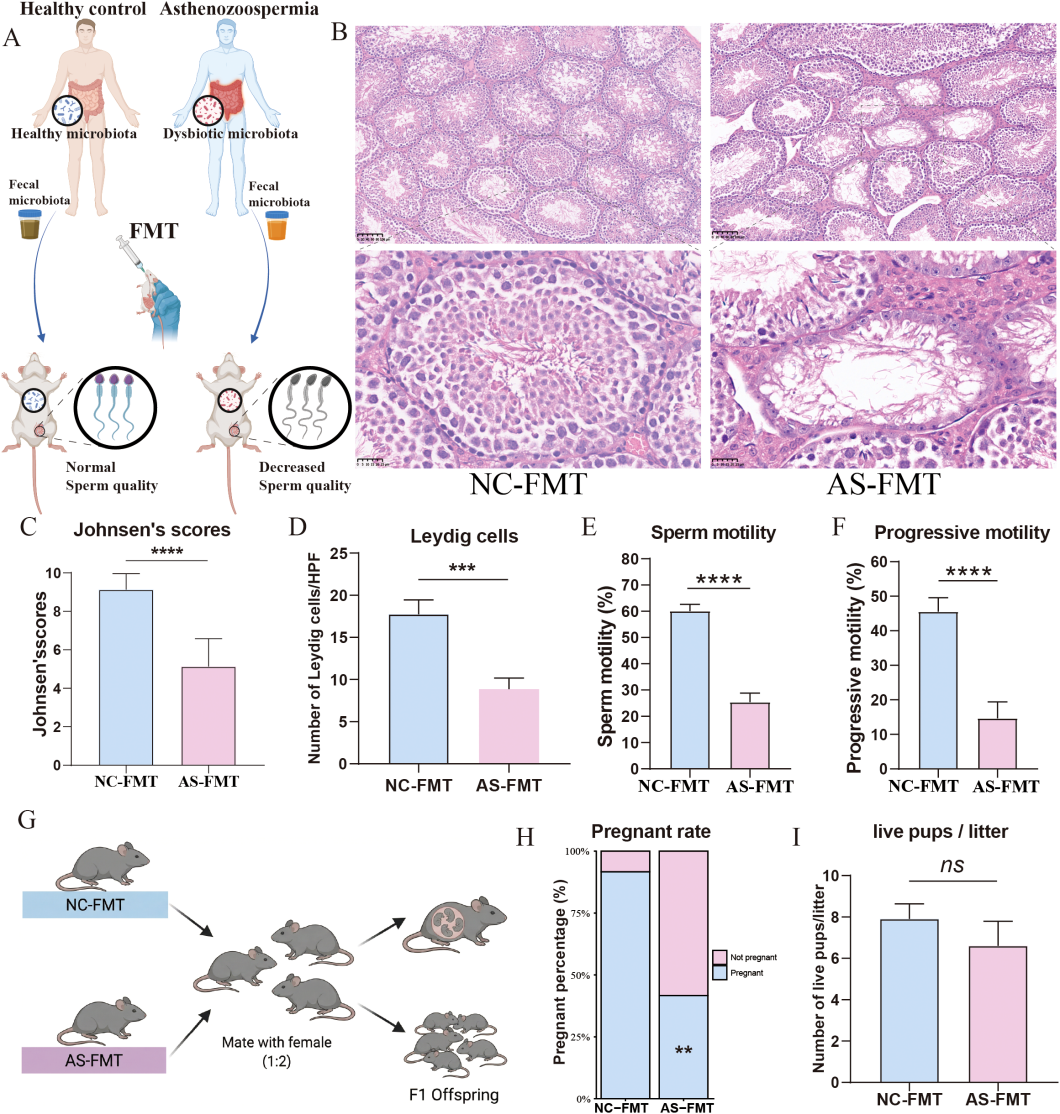
Fecal microbiota transplantation (FMT) demonstrates that the gut microbiota modulates sperm quality in mice. Transplantation of microbiota from asthenozoospermia patients led to a significant reduction in sperm quality in male recipient mice and a marked increase in abnormal seminiferous tubules within the testes. **(A)** Schematic of the FMT experimental design; **(B)** Representative hematoxylin and eosin (H&E)-stained testicular sections; **(C)** Histological evaluation of spermatogenesis using Johnsen’s mean testicular biopsy score (MTBS). Fifty seminiferous tubules were randomly analyzed per biological replicate in a blinded manner. **(D)** The number of Leydig cells. Five random high-power fields (400×) were selected for each mouse. The number of cells with typical Leydig morphology (round nuclei and eosinophilic cytoplasm) in the interstitial space was counted. **(E)** Total motility of cauda epididymal sperm; **(F)** Progressive motility of cauda epididymal sperm; **(G)** Experimental scheme for cage mating of NC-FMT and AS-FMT males with females. **(H)** Pregnancy rate and **(I)** offspring counts. FMT, fecal microbiota transplantation; NC-FMT, mice receiving microbiota from healthy controls; AS-FMT, mice receiving microbiota from asthenozoospermia patients. **, P<0.01; ***, P<0.001; ****, P<0.0001.

In FMT experiments, bacterial suspensions from patients with asthenozoospermia and healthy controls were separately transferred into germ-free mice (**Figure 3A**). Histological examination using H&E staining revealed significant structural disruptions in the testes of AS-FMT mice. Unlike the intact and well-organized seminiferous epithelium observed in the NC-FMT group, the AS-FMT group exhibited heterogeneous pathological changes (**Figure 3B**). While some tubules retained germ cells, many displayed disorganized epithelium, sloughing of germ cells into the lumen, and a marked reduction in spermatogenic cell layers. To quantify these histological defects, we applied Johnsen’s scoring system. The AS-FMT mice showed a significantly reduced Johnsen’s score compared to the NC-FMT controls, indicating a partial arrest of spermatogenesis and loss of germ cells (**Figure 3C**). The number of Leydig cells was significantly decreased in the AS-FMT group compared to the NC-FMT group (**Figure 3D**). Furthermore, transplantation of the asthenozoospermia-derived microbiota led to significant reductions in sperm motility (**Figure 3E**) and progressive motility (**Figure 3F**) in recipient males. Cage mating of partial NC-FMT and AS-FMT males were performed with females (1:2) for 7 days (**Figure 3G**). The fertility of the recipient mice was significantly impaired in the AS-FMT group compared to the NC-FMT group. Specifically, the pregnancy rate was decreased in the AS-FMT group (**Figure 3H**). However, among the successful pregnancies, the average litter size was not significantly different between the AS-FMT group and the NC-FMT group (**Figure 3I**).

### 3.4 FMT confirms that microbiota from patients with asthenozoospermia alters butyrate metabolism in recipient mice

**Figure 4:**
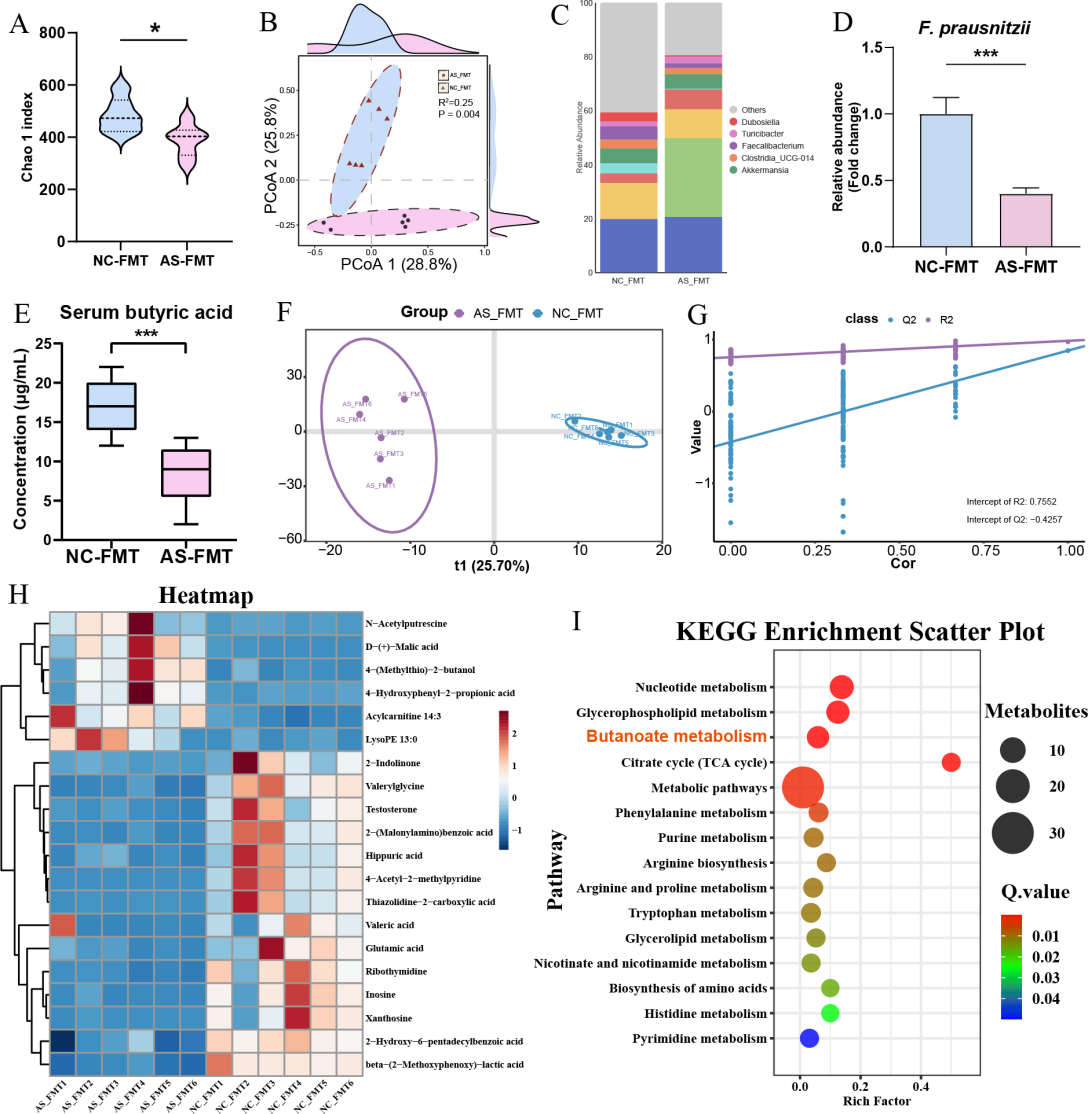
Dysbiosis in recipient mice gut microbiota and perturbation of testicular butyrate metabolism following FMT. **(A–D)** Analysis of gut microbiota in recipient mice (NC-FMT *vs*. AS-FMT): **(A)** Violin plot of the Chao1 alpha diversity index, showing significantly reduced richness in the AS-FMT group. **(B)** Principal Coordinate Analysis (PCoA) plot based on Bray-Curtis distances, demonstrating distinct clustering of the gut microbiota structure between groups (R^2^ = 0.25, *P* = 0.004, PERMANOVA). **(C)** Stacked bar plot of relative bacterial abundance at the genus level. Note the marked depletion of *Faecalibacterium* in the AS-FMT group. **(D)** Validation of the relative abundance of *Faecalibacterium prausnitzii* by qPCR, normalized to the NC-FMT group. **(E)** Quantification of serum butyrate levels in recipient mice following 8 weeks of FMT. **(F-I)** Untargeted metabolomic analysis of testicular tissues: **(F)** Score plot of the Orthogonal Partial Least Squares-Discriminant Analysis (OPLS-DA) showing distinct metabolic profiles. **(G)** Permutation test (n = 200) validating the OPLS-DA model. **(H)** Hierarchical clustering heatmap of the top 20 differential metabolites (red: high; blue: low). **(I)** Bubble plot of KEGG metabolism related pathways enrichment analysis for testicular differential metabolites, highlighting Butanoate metabolism as a top enriched pathway. *, P<0.05; ***, P < 0.001.

We next characterized the gut microbiota of the recipient mice to verify the colonization efficiency. Alpha diversity analysis indicated that the microbial richness (Chao1 index) was significantly reduced in the AS-FMT group compared to the NC-FMT group (**Figure 4A**). Principal Coordinate Analysis (PCoA) based on Bray-Curtis distances revealed a distinct separation in microbial community structure between the two groups (R^2^ = 0.25, *P* = 0.004; **Figure 4B**). Taxonomic profiling at the genus level highlighted a specific reduction in SCFA-producing bacteria, particularly *Faecalibacterium*, in the AS-FMT mice (**Figure 4C**). This reduction in *F. prausnitzii* was further validated by qPCR (**Figure 4D**). Consistent with the gut microbiome changes, metabolic profiling revealed a significant decrease in serum butyrate levels in the AS-FMT group (**Figure 4E**). Untargeted metabolomic profiling of testicular tissue from the two FMT recipient groups, analyzed by OPLS−DA, identified distinct metabolic differences (**Figures 4F, G**), yielding 140 key differential metabolites; a clustered heatmap highlights the top 20 features (**Figure 4H**). KEGG enrichment of the testicular differential metabolites indicated significant enrichment of the butanoate metabolism (butyrate metabolism) pathway (**Figure 4I**), collectively demonstrating that FMT from patients with asthenozoospermia perturbs butyrate metabolic processes in both serum and testis of recipient mice.

### 3.5 Microbiota from patients with asthenozoospermia affects testicular Leydig cells in recipient mice

**Figure 5:**
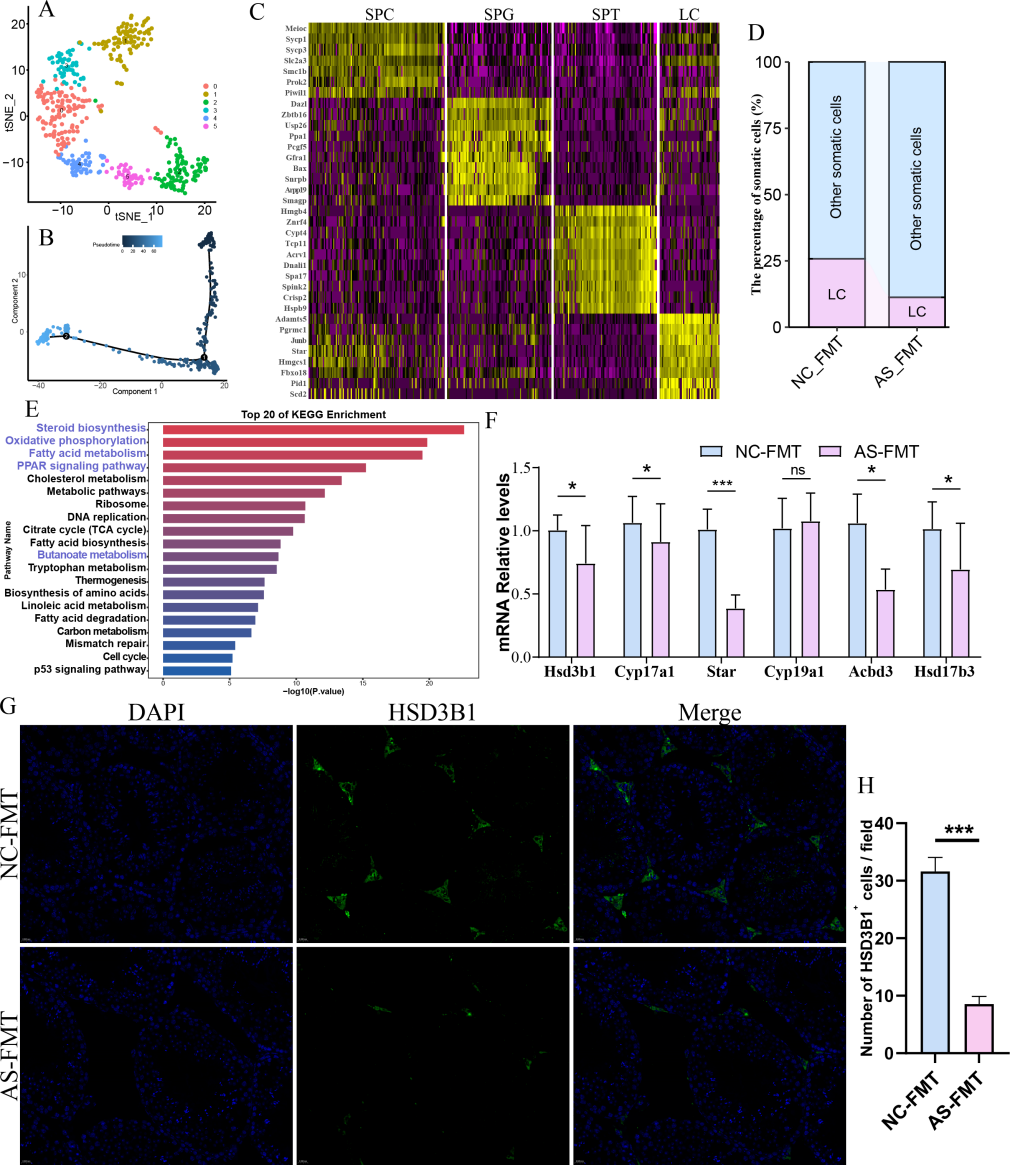
Single-cell RNA sequencing analysis of testicular tissues from mice after FMT. **(A)** t-SNE plot showing cell clustering results of core cell populations; **(B)** Pseudotime trajectory analysis; **(C)** Heatmap showing differential expression of marker genes across different cell populations; **(D)** Stacked bar plot of the relative proportions of cell populations in testicular tissues from the two groups, showing that Leydig cells (LC) exhibited the most pronounced change in the AS-FMT group compared with the NC-FMT group; **(E)** KEGG enrichment analysis (Top 20 terms) of differentially expressed genes in Leydig cells; **(F)** Relative mRNA expression levels of genes related to testosterone synthesis as determined by RT-qPCR. **(G)** Representative immunofluorescence images of HSD3B1 (green) in testicular sections from NC-FMT and AS-FMT mice. Nuclei were counterstained with DAPI (blue). Scale bar=20 µm. **(H)** Quantification of the number of HSD3B1-positive Leydig cells per field of view. *, P<0.05; ***, P<0.001.

Using single-cell RNA sequencing, we analyzed testicular tissues from two groups of mice (**Figures 5A–C**). Based on the expression of known marker genes, we identified several distinct cell clusters, including spermatogonia (SPG), spermatocytes (SPC), spermatids (SPT), and somatic cells such as Leydig cells (LC) and Sertoli cells (SC). Among these, compared with the NC-FMT group, the proportion of Leydig cells in the testicular tissue of the AS-FMT group showed the most prominent change (**Figure 5D**). KEGG enrichment analysis of the differentially expressed genes in Leydig cells revealed significant enrichment in the pathways of steroid biosynthesis, oxidative phosphorylation (OXPHOS), PPAR signaling, and fatty acid metabolism (**Figure 5E**). RT-qPCR results further demonstrated that, compared with the NC-FMT group, the relative mRNA expression levels of genes associated with testosterone synthesis were significantly decreased in the testicular tissue of the AS-FMT group (**Figure 5F**). To further validate the impact of gut dysbiosis on the testicular interstitium, we performed immunofluorescence staining for HSD3B1, a key steroidogenic enzyme and specific marker for Leydig cells. The Leydig cells in the NC-FMT group were abundant and densely packed within the interstitial spaces. In contrast, the AS-FMT group exhibited a marked reduction in HSD3B1-positive signals, with fewer visible Leydig cells in the interstitial regions (**Figure 5G**). Quantitative analysis confirmed that the number of HSD3B1-positive Leydig cells per field was significantly lower in the AS-FMT mice compared to the NC-FMT controls (**Figure 5H**). The histological findings are consistent with the scRNA-seq data, providing robust evidence that the transplanted gut microbiota from asthenozoospermia patients induces Leydig cell loss and impairs the structural integrity of the testicular interstitium. These findings suggest that FMT from patients with asthenozoospermia primarily affects testicular Leydig cells, thereby regulating testosterone synthesis and consequently influencing sperm quality in recipient male mice.

### 3.6 Gut microbiota metabolite butyrate plays a crucial role in regulating sperm quality in asthenozoospermia

**Figure 6:**
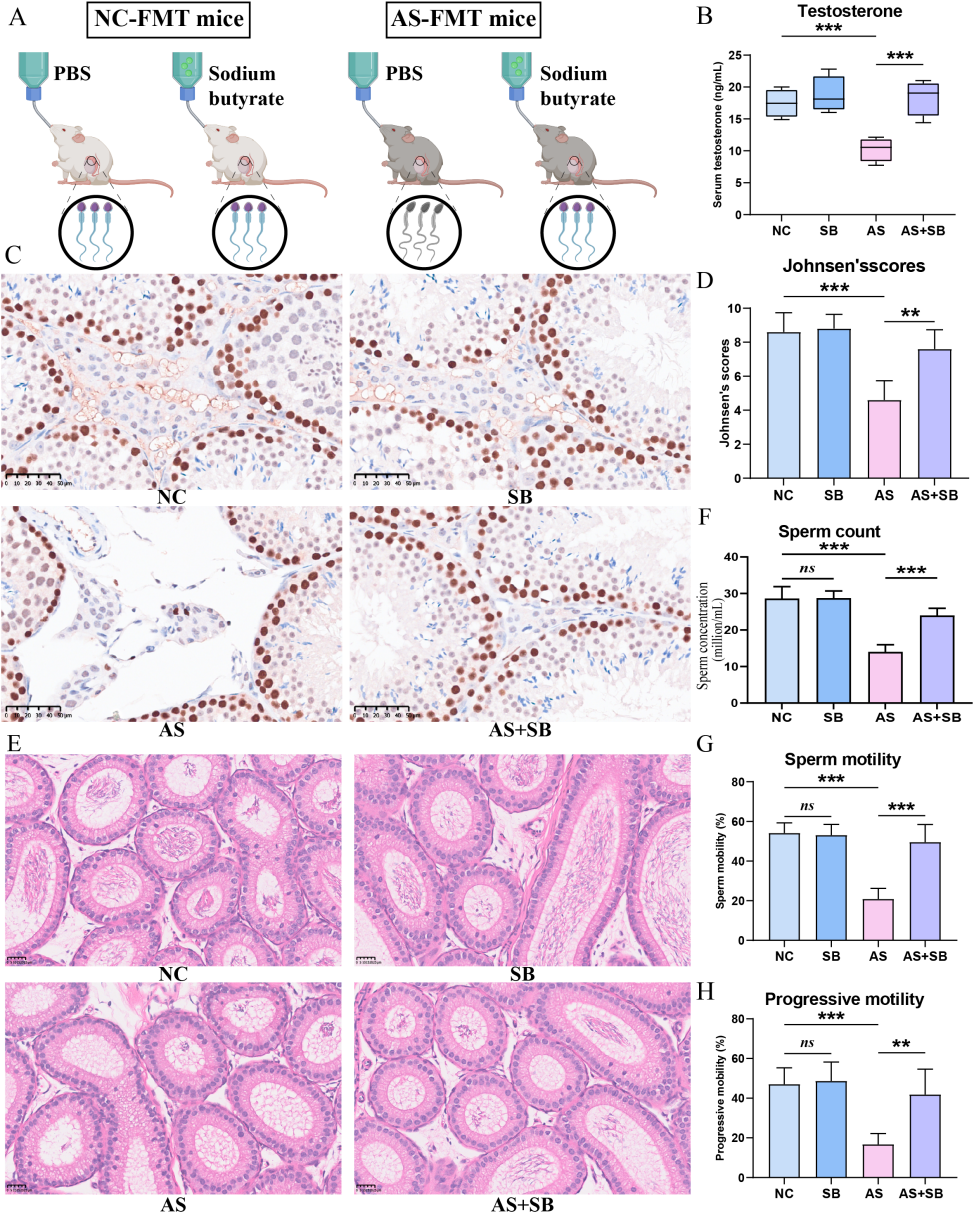
Butyrate improves sperm quality and testosterone levels in humanized FMT model male mice. A humanized FMT mouse model was established, and butyrate treatment was administered to the model mice, resulting in significant improvements in sperm quality and serum testosterone levels. **(A)** Schematic diagram of the experimental design for butyrate intervention; **(B)** Serum testosterone levels in mice; **(C)** Immunohistochemical analysis of PCNA expression in mouse testicular tissue. **(D)** Histological evaluation of spermatogenesis using Johnsen’s mean testicular biopsy score (MTBS). The reduced Johnsen’s scores induced by AS group were significantly restored in the AS+SB group. **(E)** Representative photomicrographs of hematoxylin and eosin (H&E)-stained epididymis tissue sections. Scale bar=25μm. **(F–H)** Comparison of sperm counts **(F)**, total sperm motility **(G)** and progressive motility **(H)** in the cauda epididymis; NC, the NC-FMT plus PBS group; SB, the NC-FMT plus SB (sodium butyrate) group; AS, the AS-FMT plus PBS group; AS+SB, the AS-FMT plus SB group. **, P<0.01; ***, P<0.001.

To further explore the role and potential mechanisms of the gut microbiota-derived metabolite butyrate in regulating sperm quality in male mice, we supplemented mice with either control or sodium butyrate solution concurrently with fecal microbiota transplantation (**Figure 6A**). Serum testosterone levels were significantly decreased in the AS-FMT group compared to controls (**Figure 6B**), and butyrate supplementation restored these levels. Immunohistochemical analysis of PCNA expression in testicular tissues revealed that PCNA levels in the testes were markedly reduced in model mice, whereas butyrate supplementation significantly restored PCNA expression in the testes (**Figure 6C**). At the histological level, we evaluated spermatogenesis quality using Johnsen’s mean testicular biopsy score (MTBS). While the AS group exhibited significantly lower Johnsen’s scores indicating spermatogenic arrest, the AS+SB group showed a marked improvement in scores, suggesting the recovery of structured spermatogenesis (**Figure 6D**).

Given that the epididymis is the critical site for sperm maturation and storage, we further evaluated its histological status. The cauda epididymis of the NC group displayed normal morphology with a lumen full of spermatozoa. Conversely, the AS group showed a sparse accumulation of sperm within the lumen, reflecting the impaired spermatogenic output from the testis. Notably, SB supplementation significantly ameliorated this phenotype, resulting in a dense repopulation of spermatozoa in the epididymal lumen (**Figure 6E**). In line with these pathological findings, the CASA results showed that sodium butyrate supplementation significantly improved sperm counts, sperm total motility, and progressive motility in the model mice (**Figures 6F–H**). To verify the specificity of this effect, we also evaluated the potential impact of other major SCFAs including acetate acid (AA) and propionate acid (PA). In contrast to the robust efficacy of SB, neither AA nor PA supplementation resulted in any significant improvement in sperm total motility or progressive motility compared to the AS group (**Supplementary Figures S1**), showing that the therapeutic benefit is specific to butyrate rather than a general feature of SCFA supplementation. These findings indicate that the gut microbiota metabolite butyrate plays a crucial role in regulating sperm quality in asthenozoospermia, and its mechanism of action is likely mediated primarily through the modulation of testosterone synthesis in testicular Leydig cells.

## 4 Discussion

In this study, we recruited patients with asthenozoospermia and healthy controls and collected semen, fecal, and serum samples. Metagenomic sequencing was used to characterize taxonomic and functional alterations of the gut microbiota in fecal specimens, and metabolomics was employed to quantify serum metabolite levels. We identified the key gut taxon F. prausnitzii and its metabolite butyrate, and analyzed their correlations with semen quality. We further established a humanized FMT mouse model by preparing fecal suspensions from asthenozoospermia patients and healthy controls and transplanting them into germ-free mice. We evaluated sperm quality and testosterone levels, assessed changes in testicular architecture, and measured expression changes in genes and proteins related to spermatogenesis and testosterone biosynthesis. Using metagenomic sequencing and metabolomics, we profiled key taxa and microbial functions as well as metabolite alterations, and integrated these data with findings from clinical specimens to delineate the relationships between the key gut microbiota and their metabolites and sperm quality, along with the potential pathways involved. We integrated multi-omics analyses with humanized mouse models to unravel the causal role of gut microbiota dysbiosis in the pathogenesis of asthenozoospermia. We demonstrate that asthenozoospermia is characterized by a specific depletion of butyrate-producing bacteria, notably F. prausnitzii, leading to a systemic deficit in circulating butyrate. Mechanistically, we identify a novel “gut-butyrate-testis” axis wherein butyrate functions as a pivotal signaling molecule that sustains testosterone synthesis in Leydig cells. These findings not only provide a mechanistic explanation for the metabolic regulation of spermatogenesis but also highlight the therapeutic potential of targeting the gut microbiota-butyrate axis to restore male fertility.

The gut microbiota constitutes the largest and most complex microbial ecosystem in the human body and has been shown in prior studies to regulate host health through multiple mechanisms, including modulation of metabolic activities and immune responses (16, 17). Clinical investigations at the Cleveland Clinic have revealed marked alterations in gut microbial profiles among men with infertility, and key differential genera such as Anaerococcus are associated with male fertility (9). Investigators in China have reported that the genera Bacteroides and Prevotella are associated with sperm motility in patients with abnormal semen parameters; furthermore, animal experiments demonstrated that gut dysbiosis can induce endotoxemia in male mice, increase epididymal inflammation, and reduce the testicular expression of genes related to mitochondrial function and meiosis, thereby impairing sperm quality (18). Gut dysbiosis can also lower bile acid levels, disrupt vitamin A uptake in testicular tissue, and compromise spermatogenesis, underscoring the pivotal role of the gut-testis axis in the regulation of male reproductive function (19). Our team analyzed and compared the gut, seminal, and urinary microbiota of patients with various abnormal semen parameters and found that the gut microbiota had the greatest impact on semen quality, followed by the seminal microbiota, whereas the urinary microbiota exerted a smaller effect (20). Collectively, these findings indicate that the gut microbiota can influence sperm quality and male fertility.

Asthenozoospermia is among the most common causes of male infertility. Our study revealed that patients with asthenozoospermia exhibit significant differences in gut microbiota diversity and taxonomic composition compared with healthy controls; notably, the abundance of F. prausnitzii, a butyrate-producing species, was markedly reduced, and this species showed a positive correlation with sperm motility. Our study identified F. prausnitzii as a key bacterium associated with asthenozoospermia. It is important to note that butyrate production in the gut is a collective function performed by a functional guild of bacteria, including genera such as Roseburia, Eubacterium, Anaerostipes, Coprococcus, and Subdoligranulum (21, 22). Although some such as Roseburia showed a decreasing trend in our microbiome analysis, F. prausnitzii exhibited the most significant reduction in abundance and the strongest correlation with sperm motility parameters in our cohorts. Given that F. prausnitzii is one of the most abundant butyrate producers in the human gut, its depletion likely represents the primary driver of the butyrate deficit observed in these patients (23, 24). Therefore, we focused on F. prausnitzii for further functional validation, while not ruling out the collective contribution of other butyrate-producing species to the overall gut microenvironment.

FMT involves introducing a processed fecal suspension collected from donors into the recipient’s gastrointestinal tract to directly alter the recipient’s gut microbiota with the aim of preventing, treating, or inducing disease (25). In scientific research, FMT experiments can elucidate causal relationships between microbes and disease and represent an important approach for screening and investigating the in vivo functions of microorganisms (26). To verify the impact of the gut microbiota on sperm quality and establish causality, we generated humanized FMT mice by preparing fecal suspensions from patients with asthenozoospermia and healthy controls and transplanting them into germ-free mice. We found that, compared with mice receiving microbiota from healthy controls, mice colonized with microbiota from asthenozoospermia patients exhibited significantly lower sperm motility, indicating that the gut microbiota of asthenozoospermia patients can influence sperm quality. In this study, we employed FMT by introducing microbial suspensions prepared from human fecal specimens into the gastrointestinal tracts of germ-free mice. We then assessed post-transplantation changes in host physiological, metabolic, and immunological parameters, thereby elucidating the role of the gut microbiota in phenotypic manifestation. The FMT approach is highly flexible and specific, enabling direct demonstration of causal relationships between the gut microbiota and host phenotypes under controlled experimental conditions, and providing a robust scientific basis for personalized medicine and microbiome-targeted therapeutic interventions (25, 27). Beyond validating the effects of specific microbial communities on human health, FMT can also reconstitute a healthy gut microbiota and thus represents a promising strategy for the treatment of human diseases (28). Therefore, we have reason to posit that the gut microbiota plays an important role in the onset and progression of asthenozoospermia.

The gut microbiota primarily regulates human metabolic, immune, and endocrine physiology and pathophysiology through its own metabolites and derivatives (29, 30). Metabolomic analysis enables the detection of changes in specific small-molecule metabolites in vivo and facilitates an in-depth understanding of disease-related metabolic pathways and mechanisms (31). In our study, untargeted metabolomic profiling of serum samples from patients with asthenozoospermia and healthy controls identified 39 key differential metabolites; KEGG enrichment analysis of these metabolites indicated significant enrichment of the butyrate metabolism pathway. We further conducted targeted quantification of serum short-chain fatty acids and found that serum butyrate levels were markedly reduced in patients with asthenozoospermia. Similarly, in humanized FMT mice, serum butyrate concentrations were also significantly decreased in recipients colonized with microbiota from asthenozoospermia patients compared with those receiving microbiota from healthy controls. In addition, untargeted metabolomics of testicular tissue from the two groups of FMT recipient mice, followed by KEGG enrichment analysis of the differential metabolites, likewise revealed significant enrichment of the butyrate metabolism pathway. Importantly, mammalian hosts lack the specific enzymatic machinery required for endogenous butyrate synthesis. Consequently, the butyrate detected in the serum and testis is intrinsically derived from gut microbial fermentation. Thus, the synchronous reduction of butyrate-producing bacteria in the gut and butyrate levels in the circulation and testis provides compelling evidence that the observed metabolic alterations originate from the gut microbiota. These findings suggest that butyrate may be a key metabolite mediating the effect of the gut microbiota on sperm quality in patients with asthenozoospermia.

It remains to be elucidated whether butyrate affects spermatogenesis or post-maturation motility. Our finding of altered Leydig cell function and testosterone levels suggests a primary impact on spermatogenesis, but direct effects on mitochondrial function in mature sperm cannot be excluded. As an important metabolite of the gut microbiota, butyrate is one of the short-chain fatty acids predominantly produced in the colon through microbial fermentation of undigested dietary fiber (32). It serves as a principal energy substrate for colonocytes, can be absorbed into the bloodstream via the intestine to exert systemic effects, and is closely linked to host health (13, 33, 34). Butyrate acts through multiple pathways: it engages G protein-coupled receptors (GPCRs) to inhibit neutrophil secretion of inflammatory cytokines (35); it also functions as a histone deacetylase (HDAC) inhibitor to modulate B-cell activity, increase levels of the anti-inflammatory cytokine IL-10, and reduce IL-17 expression (36). In addition, as an HDAC inhibitor, butyrate can remodel gene expression, suppress tumor cell proliferation, and induce tumor cell apoptosis, thereby exerting antitumor effects (21). Consequently, butyrate plays a critical role in maintaining intestinal health, regulating immune homeostasis, and preventing disease, representing a key nexus of host–microbiota interactions.

Furthermore, the mechanism by which butyrate restores Leydig cell function and spermatogenesis may be closely linked to its immunomodulatory properties. The testis is an immune-privileged organ, and its function is highly sensitive to inflammatory signals (37). Gut dysbiosis is known to increase intestinal permeability and elevate systemic pro-inflammatory cytokines, which can disrupt the blood-testis barrier and impair Leydig cell steroidogenesis (38–41). Butyrate is a HDAC inhibitor and acts as a potent anti-inflammatory agent (42). It has been shown to suppress nuclear factor-kappa B activation and reduce the production of pro-inflammatory cytokines (22). Therefore, the protective effect of butyrate observed in our study likely involves the mitigation of inflammation-induced testicular damage, highlighting the critical role of the gut-immune-testis axis in male reproductive health (43).

A central question in defining the “gut-testis axis” is distinguishing between immune-mediated damage like endotoxemia and the loss of beneficial metabolic signaling. The “gut-testis axis” is a sophisticated bidirectional communication system (44–46). Our study identifies a specific metabolic branch of this axis (the butyrate-Leydig cell axis), but this likely operates within a larger, multi-dimensional network. The initiation of this axis begins with the disruption of the intestinal microecosystem. The depletion of F. prausnitzii reduces butyrate production. Butyrate, as a small-molecule metabolite, enters the portal circulation and reaches the testis via systemic blood flow. Our results support this direct action, as butyrate directly restored Leydig cells number and function. Previous studies have shown that gut dysbiosis can trigger systemic low-grade inflammation (47, 48). This systemic “cytokine storm” or immune cell trafficking could impair the blood-testis barrier or induce oxidative stress in the testicular microenvironment (49, 50). Earlier research has heavily emphasized the “endotoxemia-inflammation” model, where gut-derived LPS induces testicular inflammation (18). We propose that our “metabolic-steroidogenic” model is complementary and synergistic to the inflammatory model. Under different pathophysiological conditions, such as acute infection versus chronic dietary-induced dysbiosis, one pathway may dominate over the other, but both converge on the disruption of spermatogenesis. This comprehensive map clarifies that the butyrate signaling identified in our work is a vital, metabolic “tether” that maintains the endocrine stability of the gut-testis axis.

The present study has several limitations. First, the clinical samples in this study were obtained from a single center, which may introduce selection bias; subsequent validation in large, multicenter cohorts is required. Second, for the mouse FMT experiment, we used whole fecal materials that included bacteria, fungi, virus, phages, proteins and metabolites from both bacteria and the host. Therefore, it is not precisely clear which component from the feces functions in asthenozoospermia. It is important to use multi-omics techniques, inactivated feces and isolated bacteria/metabolites to affirm the key factors contributing to asthenozoospermia. Third, this study suggests that a gut microbiota derived metabolite butyrate may be a key regulator of sperm quality in asthenozoospermia. The mode of action of butyrate in modulating asthenozoospermia including its principal target cells and molecular mechanisms needs to be validated. Fourth, we analyzed serum metabolomics rather than seminal plasma. While seminal plasma directly reflects the sperm microenvironment, serum profiling provides insight into the systemic metabolic crosstalk driven by the gut microbiota. Future studies should prioritize paired seminal plasma analysis to confirm local metabolic shifts.

Our study has some excellent strengths and advantages. First, our study integrated clinical specimens, germ-free mice, FMT, and in vivo experiments to investigate the effects and specific roles of the gut microbiota and its metabolites on asthenozoospermia, and employed multiple molecular biology techniques to elucidate the underlying mechanisms linking the gut microbiota and its metabolites to sperm quality. To our knowledge, no similar comprehensive study has been reported in the literature. Second, we identified the important role of alterations in the gut microbiota and its metabolite butyrate in the asthenozoospermia, thereby advancing mechanistic understanding of the disease from the perspective of the microbiota and its metabolites and providing new avenues for therapy. Third, this clinically driven, innovative study paves the way for subsequent clinical trials to evaluate the therapeutic efficacy of interventions such as supplementation with probiotics containing butyrate-producing bacteria or dietary enrichment with butyrate-rich foods for the treatment of clinical asthenozoospermia-approaches characterized by a favorable safety profile and strong feasibility.

## 5 Conclusions

Overall, we identified here obvious gut dysbiosis in patients with asthenozoospermia and proved that this dysbiosis can cause changes in sperm quality and testosterone levels. The possible underlying mechanisms are that dysbiosis influences the host’s metabolites and causes local testicular dysfunction. In conclusion, our study indicates a microbiota-metabolite-testis axis whereby F. prausnitzii derived butyrate maintains Leydig cell steroidogenic function, offering a promising metabolic target for treating asthenozoospermia.

## Data Availability

The datasets presented in this study can be found in online repositories. The names of the repository/repositories and accession number(s) can be found in the article/**Supplementary Material**.
