# Supplementary material for: Gut dysbiosis induces the development of asthenozoospermia through butanoate metabolism
Source: Front Immunol. 2026 Mar 18;17:1760881. doi: 10.3389/fimmu.2026.1760881 (PMC13038517; doi:10.3389/fimmu.2026.1760881)
Supplement: Supplementary file 1 [file DataSheet1.pdf]

Supplementary Material

Supplementary Materials and Methods

1. Shotgun metagenomic sequencing

Each participant provided a single fresh stool sample for shotgun metagenomic sequencing. Total microbial DNA was extracted using the Cetyltrimethylammonium bromide (CTAB) method. Total genomic DNA was also extracted from mouse cecal contents using CTAB method. DNA extraction quality was assessed by agarose gel electrophoresis. DNA concentration was quantified with an ultraviolet (UV) spectrophotometer. Shotgun metagenomic sequencing was performed on the Illumina NovaSeq 6000 platform as 2 x 150 bp paired-end reads. Trimmomatic v_0.39 was used to trim low-quality base and short length reads using the following parameters: SLIDINGWINDOW:4:30 and MINLEN:100. Bowtie2 (version 2.3.5) was used to discard reads from the human genome. Taxonomic assignment was performed on high-quality metagenomics reads using k-mer based algorithms in Kraken2 pipeline with default settings. Bracken was used to re-estimate a more accurate sequence abundance at genus or species levels. Rarefaction by subsampling was performed on final metagenomic read counts to account for differences in sequencing depth. Alpha diversity was evaluated using the Chao1 index to assess microbial richness. Beta diversity was visualized using Principal Coordinate Analysis (PCoA) based on Bray-Curtis dissimilarity matrices, and statistical differences in community structure were assessed by Permutational Multivariate Analysis of Variance (PERMANOVA). Taxonomic microbial composition was analyzed at the genus level, and relative abundance was visualized using stacked bar charts. The Linear Discriminant Analysis Effect Size (LEfSe) was performed to compare abundances of all bacterial clades between AZS patients and healthy controls. Linear discriminant analysis (LDA) score bar plot was used to show the species meeting a significant LDA threshold value of >3 and False Discovery Rate (FDR)-adjusted $P<0.05$ and these taxa were considered as significantly differential species.

2. Untargeted metabolomics analysis

Sample preparation was performed as previously described with minor modifications.

Quality control (QC) samples were prepared by pooling equal aliquots of all samples and injected at regular intervals to monitor system stability. Briefly, 100 µL of human serum or 50 mg of mouse testicular tissue was mixed with 400 µL of cold extraction solvent (methanol: acetonitrile: water = 2:2:1, v/v/v), containing isotopically-labeled internal standards. Testicular tissues were homogenized using a tissue lyser. The mixture was vortexed for 30 s, sonicated for 10 min at 4°C, and incubated for 1 h at -20°C to precipitate proteins. After centrifugation at 14,000 × g for 20 min at 4°C, the supernatant was collected and dried in a vacuum centrifuge. The dried samples were reconstituted in 100 µL of acetonitrile: water (1:1, v/v) for LC-MS/MS analysis.

Chromatographic separation was performed using a Vanquish UHPLC system (Thermo Fisher Scientific) equipped with an ACQUITY UPLC BEH Amide column (2.1 mm × 100 mm, 1.7 µm). The mobile phase consisted of (A) 25 mM ammonium acetate and 25 mM ammonium hydroxide in water and (B) acetonitrile. The gradient elution program was set as follows: 0-0.5 min, 95% B; 0.5-7.0 min, 95% to 65% B; 7.0-8.0 min, 65% to 40% B; 8.0-9.0 min, 40% B; 9.0-9.1 min, 40% to 95% B; and 9.1-12.0 min, 95% B for column re-equilibration. The flow rate was maintained at 0.3 mL/min, and the column temperature was kept at 25°C.

Mass spectrometry data were acquired using a Q Exactive HF-X mass spectrometer (Thermo Fisher Scientific) in both positive and negative ionization modes. The raw data were processed using Compound Discoverer 3.1 for peak alignment, normalization, and metabolite identification against the HMDB database. For multivariate statistical analysis, the normalized data matrix was imported into SIMCA software. Unsupervised Principal Component Analysis (PCA) was first employed to visualize the overall distribution and detect outliers. Subsequently, supervised Orthogonal Partial Least Squares-Discriminant Analysis (OPLS-DA) was performed to maximize the separation between the AZS and healthy control groups and to identify differential metabolic features. The robustness of the OPLS-DA model was validated using a 200-permutation test to prevent overfitting (R^2 and Q^2 values were assessed). Differential metabolites were screened based on the Variable Importance in Projection (VIP) scores obtained from the OPLS-DA model and the *P*-values from the Student's t-test. Metabolites with VIP > 1.0 and $P<0.05$ were considered significantly altered.

To explore the biological significance of the identified differential metabolites, pathway enrichment analysis was performed using MetaboAnalyst 6.0 (https://www.metaboanalyst.ca). The standardized HMDB IDs were mapped to the Kyoto Encyclopedia of Genes and Genomes (KEGG) pathway database (Homo sapiens library). Enrichment analysis was based on the Global Test algorithm, and pathway topology analysis used Relative Betweenness Centrality. Pathways with a FDR adjusted P<0.05 were considered significantly enriched.

3. Targeted SCFAs metabolomics analysis

Serum concentrations of short-chain fatty acids (SCFAs), including butyrate, were quantified using a liquid chromatography-tandem mass spectrometry (LC-MS/MS) method with 3-nitrophenylhydrazine (3-NPH) derivatization. Briefly, 20 μL of serum sample or standard solution was mixed with 100 μL of 50% aqueous acetonitrile. Then, 40 μL of 200 mM 3-NPH (in 50% aqueous acetonitrile) and 40 μL of 120 mM N-(3-dimethylaminopropyl)-N′-ethylcarbodiimide hydrochloride (EDC) containing 6% pyridine were added to the mixture. The reaction was incubated at 40°C for 30 min to convert carboxylic acids into stable hydrazides. After incubation, the mixture was diluted with dilution solvent, e.g., 10% acetonitrile and centrifuged at 12,000 × g for 10 min. The supernatant was collected for analysis. Chromatographic separation was performed on an ExionLC AD System (SCIEX) equipped with a Phenomenex Kinetex C18 column (2.1 mm × 100 mm, 1.7 μm). The mobile phases consisted of (A) 0.1% formic acid in water and (B) acetonitrile. The gradient elution was set as follows: 0-1 min, 10% B; 1-6 min, 10%-50% B; 6-10 min, 50-95% B at a flow rate of 0.3 mL/min.

Mass spectrometric detection was performed using a QTRAP 6500+ (SCIEX) in negative electrospray ionization (ESI-) mode. Quantification was carried out using the Multiple Reaction Monitoring (MRM) mode. The specific ion transitions for butyrate-3-NPH derivative and its corresponding internal standard were optimized. Data acquisition and processing were performed using Analyst 1.7.1 and MultiQuant 3.0.2.

4. Single-cell RNA sequencing (scRNA-seq) of testicular tissue

1) Tissue dissociation and library preparation

Freshly harvested mouse testes were immediately washed in ice-cold PBS. The tunica albuginea was carefully removed, and the testicular parenchyma was minced into approximately 1 mm^3 pieces. A two-step enzymatic digestion protocol was employed to maximize cell viability and diversity. First, the tissue was incubated in HBSS containing 1 mg/mL Collagenase IV and 200 μg/mL DNase I at 37°C for 15 min with gentle agitation to dissociate the interstitial tissue. Second, the seminiferous tubules were sedimented and further digested in 0.25% Trypsin-EDTA supplemented with 1 mg/mL Hyaluronidase for 10 min at 37°C to obtain a single-cell suspension. The digestion was quenched by adding equal volumes of DMEM containing 10% FBS. The resulting cell suspension was filtered through a 40-μm nylon mesh and centrifuged at 300 × g for 5 min. Cell viability was assessed using Trypan Blue staining, and only samples with >85% viability were processed. Single-cell suspensions were adjusted to a concentration of 1,000 cells/μL. Appropriate volumes of single-cell suspensions were loaded into the 10x Genomics Chromium Controller using the Chromium Single Cell 3' Reagent Kit v3.1. cDNA amplification and library construction were performed according to the manufacturer's standard protocols. Libraries were sequenced on an Illumina NovaSeq 6000 platform to achieve sufficient targeted depth per cell.

2) scRNA-seq data processing and quality control

Raw sequencing data were demultiplexed, aligned to the mouse reference genome (GRCm38), and quantified using CellRanger software (v6.1.1). The resulting feature-barcode matrices were imported into the R environment for downstream analysis using the Seurat package (v4.0). Stringent quality control (QC) metrics were applied to filter low-quality cells, empty droplets, and multiplets. QC filtering criteria were established based on the distribution of metric characteristics (nFeature_RNA, nCount_RNA, and mitochondrial percentage) to preserve biological heterogeneity while effectively removing technical artifacts. A relatively relaxed mitochondrial threshold was dynamically adapted for testicular tissue to account for the inherently high metabolic activity and mitochondrial abundance in developing germ cells. Data were normalized using the NormalizeData function (LogNormalize method), and the top 2,000 highly variable features were identified using the FindVariableFeatures function. To minimize batch effects and technical noise, data scaling was performed with the ScaleData function,

regressing out mitochondrial gene percentage.

3) Dimensionality reduction, clustering, and annotation

Principal Component Analysis (PCA) was performed on the scaled data. The top 20 principal components (PCs) were selected for non-linear dimensionality reduction using Uniform Manifold Approximation and Projection (UMAP) and t-Distributed Stochastic Neighbor Embedding (t-SNE). UMAP was executed with default parameters to preserve the global structure of the data, while t-SNE was run using the RunTSNE function to emphasize local neighborhood relationships. Unsupervised cell clustering was conducted using the FindNeighbors and FindClusters functions with optimized resolution parameter determined by the clustering stability and biological interpretability of the resulting subsets. Cell types were annotated based on the expression of canonical marker genes established in the literature. To compare the cellular composition between groups, the proportion of each cell type was calculated relative to the total number of cells per sample.

4) Pseudotime trajectory analysis

To reconstruct the developmental trajectory of germ cells, single-cell trajectories were modeled using the Monocle 2 packa The Seurat object was converted into a CellDataSet. Genes used for ordering cells were defined based on the differentially expressed genes across clusters. Dimensionality reduction was performed using the DDRTree algorithm, and cells were ordered along the trajectory based on pseudotime. The trajectory structure was visualized to reveal the continuous progression of spermatogenesis.

5) Differential expression and enrichment analysis in Leydig cells

To investigate the functional alterations in Leydig cells, differential expression analysis was performed specifically on the Leydig cell cluster between the two groups. Differentially expressed genes (DEGs) were identified using the FindMarkers function in Seurat based on the Wilcoxon Rank Sum test (adjusted $P<0.05$ and $|\log2\ FC|>0.25$). To elucidate the biological functions associated with these DEGs, Kyoto Encyclopedia of Genes and Genomes (KEGG) pathway enrichment analysis was performed using the clusterProfiler R package. P-values were adjusted for multiple comparisons using the Benjamini-Hochberg procedure, and pathways with an adjusted $P<0.05$ were considered significantly enriched.

Supplementary Tables

Table S1. Primer sequences for RT-qPCR (Mouse)

Gene	Forward Primer (5’→3’)	Reverse Primer (5’→3’)
Gapdh	AATGGATTTGGACGCATTGGT	TTTGCACTGGTACGTGTTGAT
Star	CAGCCCATGGACAGACTCTA	TCATGAGTGATGACCGTGTCT
Cyp17a1	ATCAGCTGGCCAGAGAAGTG	CGACGCCTTTTCCTTGGT
Hsd3b1	GGCATCTCTGTTGTCATCCA	TGAGCTGCAGAAGATGAAGG
Cyp19a1	GAGAGTTCATGAGAGTCTGGATCA	CATGGAACATGCTTGAGGACT
Hsd17b3	GAGGAAAGGCCTCATCTTGA	GAATAAGGGGTCAGCACCTG
Acbd3	AAAAACTGAAGTTCGTGGCACT	CTCTCCTCCTATCATTCCCCAA

Supplementary Figures

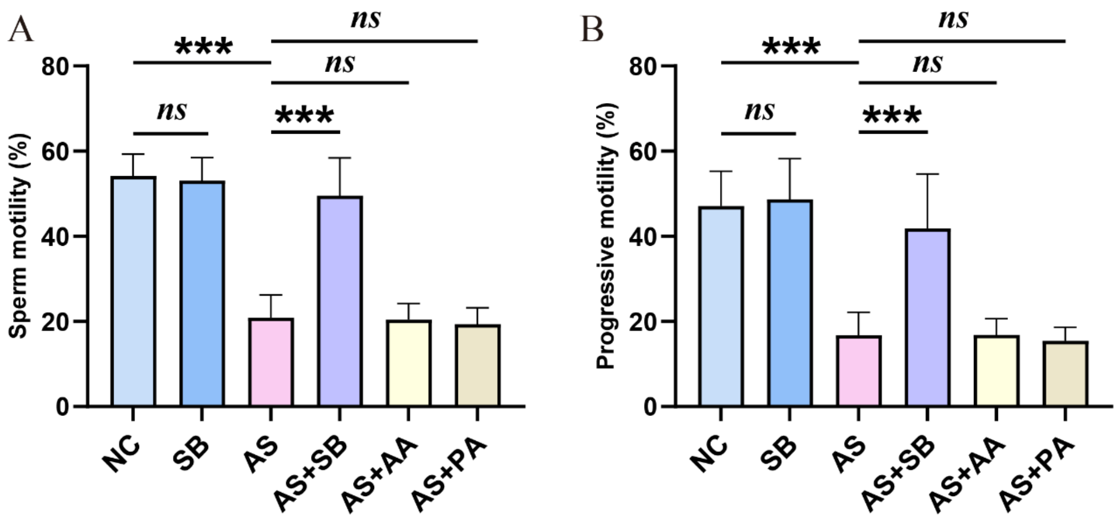

Figure S1. Specificity of butyrate in improving sperm motility. (A-B) Statistical analysis of sperm total motility (A) and progressive motility (B) in AS model mice treated with different SCFAs. Mice in the AS group were supplemented with sodium acetate acid (AA), sodium propionate acid (PA), or sodium butyrate (SB). Note that only SB treatment significantly improved sperm motility, whereas AA and PA showed no significant therapeutic effects compared to the AS model group.
